# Biomedical research in a Digital Health Framework

**DOI:** 10.1186/1479-5876-12-S2-S10

**Published:** 2014-11-28

**Authors:** Isaac Cano, Magí Lluch-Ariet, David Gomez-Cabrero, Dieter Maier, Susana Kalko, Marta Cascante, Jesper Tegnér, Felip Miralles, Diego Herrera, Josep Roca, Synergy-COPD consortium

**Affiliations:** 1 IDIBAPS-Hospital Clínic, CIBERES, Universitat de Barcelona, 08036, Barcelona, Catalunya, Spain; 2 Department of eHealth, Barcelona Digital, Roc Boronat 117, 08017 Barcelona, Catalunya, Spain; 3 Unit of Computational Medicine, Department of Medicine, Center for Molecular Medicine, Karolinska Institutet, Karolinska University Hospital, Stockholm, Sweden; 4 Biomax Informatics AG, Robert-Koch-Str. 2, Planegg, Germany; 5 Departament de Bioquimica i Biologia Molecular i IBUB, Facultat de Biologia, Universitat de Barcelona, 08028 Barcelona, Spain; 6 Almirall R&D, 08980 Sant Feliu de Llobregat, Barcelona, Spain; 7 Centro de Investigacíon Biomédica en Red de Enfermedades Respiratorias (CIBERES), Bunyola, Balearic Islands

**Keywords:** Biomedical Research, Chronic care, Clinical Decision Support Systems, Integrated Health Care Systems, Patient Decision Support Systems, Personal Health Folder

## Abstract

This article describes a Digital Health Framework (DHF), benefitting from the lessons learnt during the three-year life span of the FP7 Synergy-COPD project. The DHF aims to embrace the emerging requirements - data and tools - of applying systems medicine into healthcare with a three-tier strategy articulating formal healthcare, informal care and biomedical research. Accordingly, it has been constructed based on three key building blocks, namely, novel integrated care services with the support of information and communication technologies, a personal health folder (PHF) and a biomedical research environment (DHF-research). Details on the functional requirements and necessary components of the DHF-research are extensively presented. Finally, the specifics of the building blocks strategy for deployment of the DHF, as well as the steps toward adoption are analyzed. The proposed architectural solutions and implementation steps constitute a pivotal strategy to foster and enable 4P medicine (Predictive, Preventive, Personalized and Participatory) in practice and should provide a head start to any community and institution currently considering to implement a biomedical research platform.

## Background

The seminal purpose of the systems medicine design [1] conceived for the Synergy-COPD project [2] was to generate knowledge on underlying mechanisms explaining heterogeneities observed in chronic obstructive pulmonary disease (COPD) patients. The ultimate aim of the project was to use such knowledge to refine patient's stratification, prognosis and treatment response, which then should lead to efficient preventive strategies aiming at modulating disease progress while reducing the burden of COPD on healthcare.

**Figure 1:**
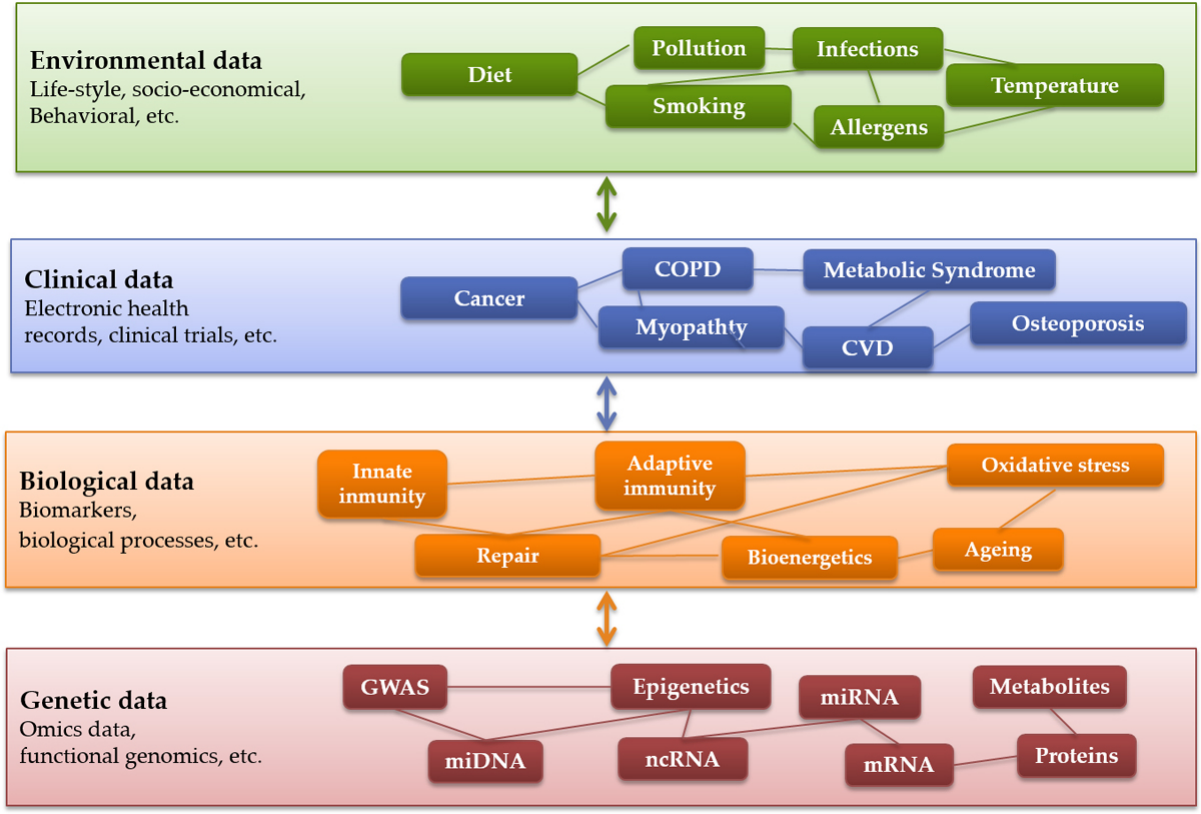
**The holistic approach intrinsic to system medicine of non-communicable diseases (NCDs) prompts the need for multilevel integration of heterogeneous patient information generated by different data sources, namely: environmental, clinical and biological data**.

It is well accepted that predictive medicine is opening entirely new and fascinating scenarios for the interplay between clinical practice and biomedical research. However, at the same time, it is generating novel requirements with impact on adoption. Firstly, the need for multilevel integration of heterogeneous patient information (Figure 1), namely: socio-economical, life-style, behavioural, clinical, physiological, cellular and "omics" data [3] and their use for the design of a personalised digital patient from virtual physiological models. Secondly, the need to extend current trends on open data from the biomedical community [4] to the clinical practice and the whole society, by engaging citizens and solving privacy and regulatory constraints, Finally, the need for highly applicable user-profiled functionalities for data management and knowledge generation. Accordingly, innovative and robust Information and Communication Technologies (ICT) will be needed as supporting tools to overcome well identified current functional limitations [5].

**Figure 2:**
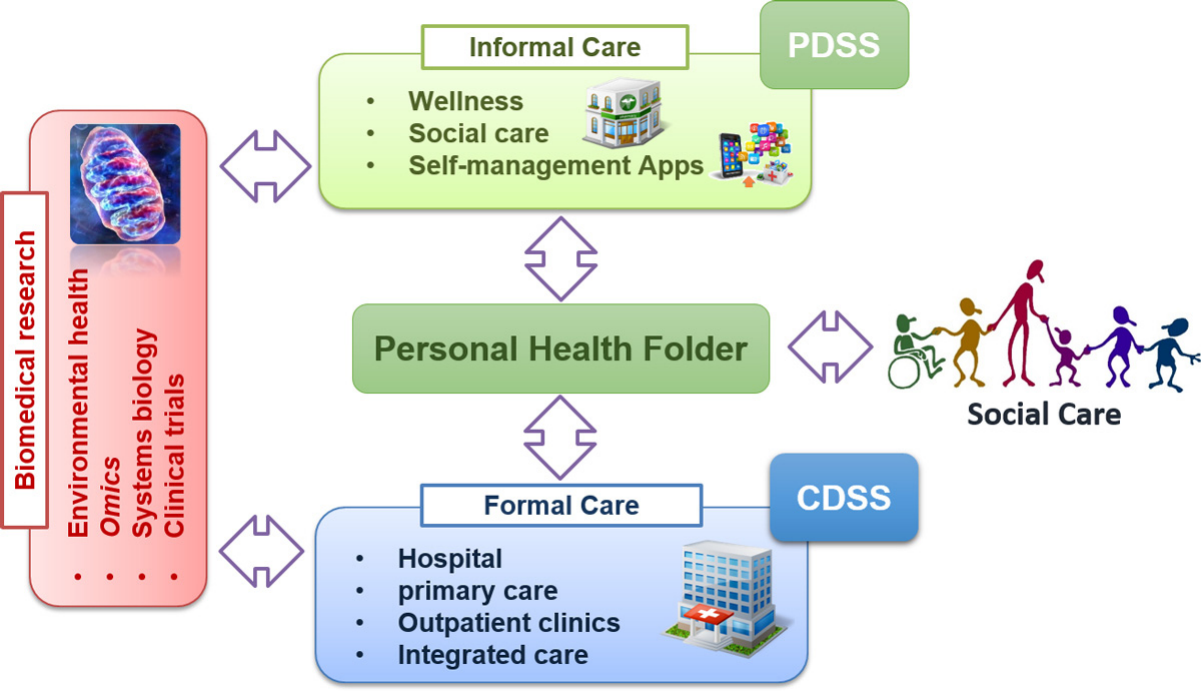
**The concept of Digital Health Framework covers the different areas wherein information can be obtained and actions are taken: *i) *informal care, *ii) *formal care, and, *iii) *biomedical research**. In this scenario, a personal health folder incorporating patient decision support systems (PDSS) might facilitate the incorporation of data coming from informal care into formal healthcare. In addition, biomedical research, referring to all research levels from clinical to basic research, should be shaped to provide user-profiled functionalities such that research professionals with different profiles can make use of clinical and biomedical knowledge from formal healthcare and heterogeneous biomedical research data sources, ultimately leading to the generation of novel rules that should feed in-place clinical decision support systems (CDSS).

The concept of **Digital Health Framework **(DHF) (Figure 2) emerged from the Synergy-COPD project to foster adoption of predictive medicine. The DHF consists of the articulation of open and modular ICT components supporting organizational interoperability, and appropriate functionalities, among three main areas, namely: *i) *informal care, *ii) *formal care, and, *iii) *biomedical research. Briefly, informal care includes any aspect with impact on health (e.g. life style, environmental and behavioural aspects, etc.) occurring in the community, whereas formal care refers to any interaction with health professionals at different levels of the healthcare system. Biomedical research refers to all research levels from bench to clinical and to public health. In our hands, to materialize such an ambitious vision, a building block approach is considered necessary. Moreover, the progress from the initial proof-of-concept to pilot implementation and to extensive deployment shall be planned following a stepwise strategy.

The first building-block was the design and initial deployment of an open source **Integrated Care Shared Knowledge Platform **supporting Integrated Care Services (ICS) for chronic patients, initiated at the EU project NEXES [6]. During the life span of NEXES, a **Personal Health Folder (PHF) **was successfully piloted [7], as a supporting tool to achieve long-term sustainability of training-induced effects and promote active life styles in COPD patients. The PHF constitutes the second building block currently being implemented in a health district (Barcelona-Esquerra, 540.000 inhabitants) in Barcelona as a tool to integrate informal and formal care in community-based ICS for frail chronic patients. Moreover, the **Clinical Decision Support Systems (CDSS) **generated during the Synergy-COPD lifetime [8] has been integrated into an ICT platform supporting ICS. Consequently, the interplay between the first two building blocks (informal and formal care) is operational in a controlled deployment scenario and is currently evolving toward maturity. We shall keep in mind, however, that active citizens/patients together with a prepared multidisciplinary health workforce will be the real key drivers of the transition from current healthcare practice to full deployment of predictive medicine for chronic patients.

The current report focuses on the third building block of the DHF, tackling biomedical research (DHF-research). The manuscript analyses architectural design, functionalities and implementation strategies of interoperability between healthcare and biomedical research.

### Progress from this paper

We report on the key elements needed for the design of the DHF-research, showing interoperability with the open Integrated Care Shared Knowledge Platform already operational in the Barcelona-Esquerra health district. From the methodological standpoint, the manuscript performs a systematic description of the DHF-research architecture and functionalities based on the lessons learnt in Synergy-COPD indicating the contributions beyond the current state of the art. The expected outcome of the manuscript is to generate a proof-of-concept of the DHF-research component as well as to propose strategies for its assessment and future implementation.

### The DHF principles: lessons learned from the Synergy-COPD project

#### Main actors of the DHF

Table 1 describes the profiles of typical actors in the DHF: biomedical research actors (Clinical and Basic scientists), healthcare actors (specialized and primary care), and three patient profiles corresponding to three possible COPD status (moderate COPD, complex COPD and COPD with comorbid conditions). Furthermore, the three patient profiles represent different levels of stratification and personalized therapeutic strategies guided by CDSS. The CDSS is embedded into an Integrated Care Shared Knowledge Platform which, in turn, make summary PHF information available to the clinician. In all three cases, the PHF-PDSS facilitates bidirectional interactions between patients and health professionals toward improving adherence to treatment, patient empowerment of his/her health status and interfacing with informal and social care [7]. Finally, the professionals with **research roles **will be using the **biomedical research platform (DHF-research)**, which has as data sources clinical information (through the Integrated Care Shared Knowledge Platform) as well as heterogeneous public and corporate sources of biomedical research data and the necessary tools to manage and process these data.

**Table 1:** Description of Actors and Roles in the Digital Health Framework

Actors		Roles	Complexity	ICT support
patient	COPD-moderate	Self-management	PC	PHF-PDSS
patient	COPD-complex	Self-management	PC&Specialist	PHF-PDSS
patient	COPD-co-morb	Self-management	PC&Specialist	PHF-PDSS
Primary Care		Clinical	Primary Care	CDSS
Specialized Care		Clinical	Secondary Care	CDSS

Clinical Scientist		Research	Academic	DHF-research
Basic Scientist		Research	Academic	DHF-research

**COPD-moderate**, patient with moderate functional impairment and low activity disease; **COPD-complex**, patient with moderate to severe functional impairment with active disease both at pulmonary and systemic levels (skeletal muscle dysfunction/wasting); **COPD-co-morb**, patient with moderate functional impairment, high activity disease and co-morbid conditions (metabolic syndrome and cardiac failure);**PC**, Primary Care professionals (doctor & nurse); **Specialized Care**, Specialized Care (doctor&nurse); **Clinical Scientist**, Translational research with clinical focus; **Basic Scientist**, Translational research with focus on basic sciences; **PHF-PDSS**, personal health folder including Patient Decision Support Systems; **CDSS**, Clinical Decision Support Systems; **DHF-research**, Digital Health Framework-Biomedical research platform.

Overall, an integrated care scenario provides support to organizational and technical interoperability between informal care and healthcare systems, which in turn feeds the DHF-research with controlled and standardized clinical information for off-line research purposes. As an example, the Synergy-COPD project identified that oxidative stress may play a central role in COPD-complex and COPD-co-morb such that research professionals (with translational clinical and basic background) cooperate using the DHF-research to further explore other poorly known mechanisms and to generate combined biomarkers to assess novel therapeutic strategies in the clinical scenario. The DHF-research should be shaped to provide user-profiled functionalities such that research professionals with different profiles can make use of it, ultimately leading to the generation of novel rules that should feed in-place CDSS and PDSS.

#### Functional principles

Based on the storyboard presented in the previous section we identified three key functional principles for the correct adoption of the DHF-research, namely: *(i) *data standardization strategies and open data policies, toward technical, syntactic and semantic interoperability between clinical data systems and biomedical research platforms, taking into account the privacy challenges of biomedical data sharing; *(ii) *profile-specific visual data mining functionalities covering the disparate needs and backgrounds of the DHF actors; and finally *(iii) *profile-specific environments supporting multi-scale predictive modelling and several forms of complex data analytics.

#### Architectural principles

The DHF-research architecture considers specific components to cover the need for organizational interoperability between research professionals working together over the same hypothesis and study design, as identified during the execution of Synergy-COPD. Moreover, general architectural principles are considered toward an open source solution with a rich set of functionalities empowering knowledge sharing, data querying and data analytics by means of a distributed, multi-layer, service oriented and ontology-driven architecture.

#### Ethical and legal issues

The evolving European legislation on health data transfer and security [9-12] was analysed and taken into account for the design of each of the three components of the DHF (Figure 2) and facilitate the empowerment of patients to open their personal clinical data and derived studies to the scientific community.

#### Deployment strategies and assessment

The three building blocks of the DHF (Figure 2) have independent deployment strategies all of them with a stepwise approach. The DHF-research is in an early phase of the design of the proof-of-concept generated from the lessons learnt in the Synergy-COPD project. Steps for a proper validation and identification of strategies for deployment were defined in the study.

### Description of the components and functionalities of the DHF-research

**Figure 3:**
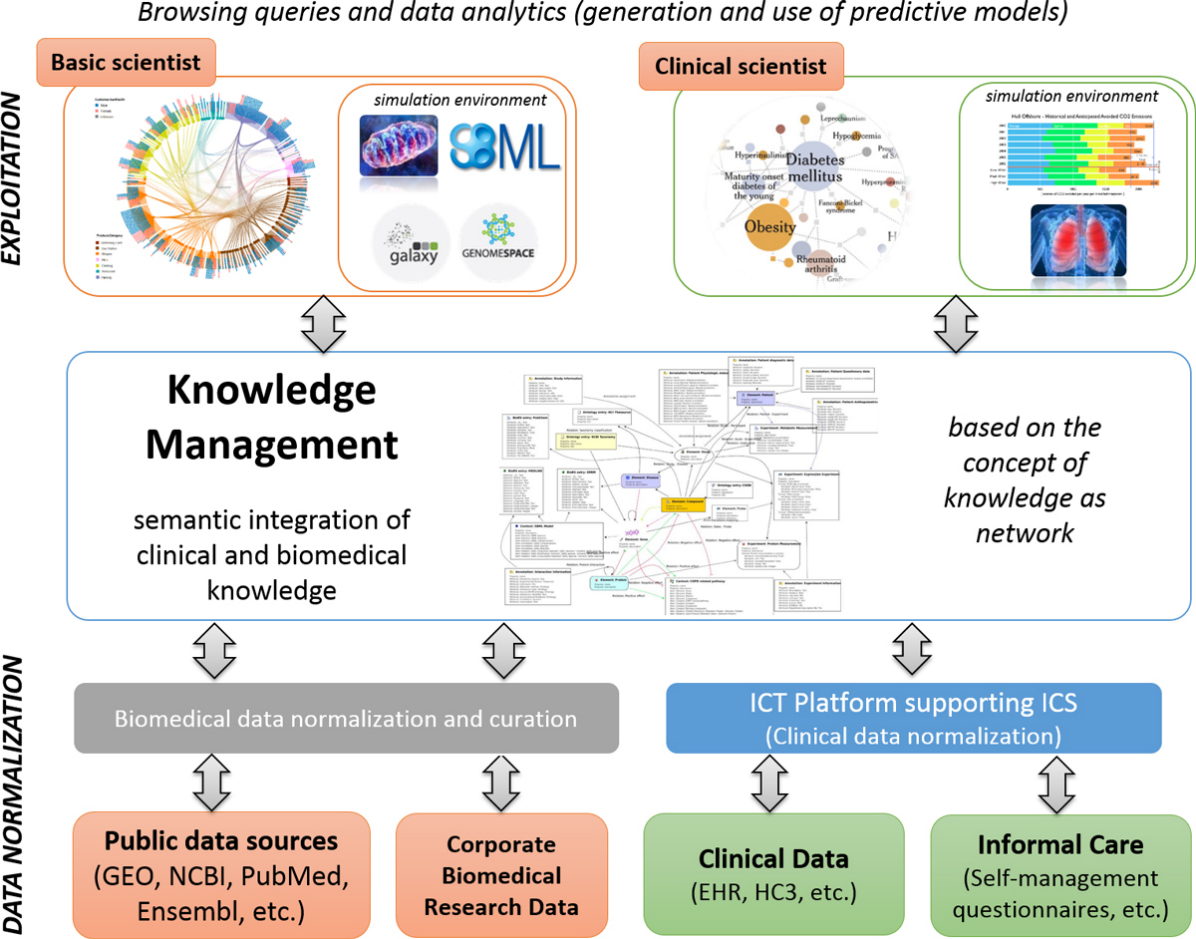
**DHF-research components and functionalities include semi-automatic data standardization approaches, data integration and knowledge management, profile-specific visual data mining portals and user-profiled simulation environments (see text for further details)**.

The Synergy-COPD project prompted the identification of three pivotal layers of the DHF-research, depicted in Figure 3, to support the following research functionalities: *i) *a semi-automatic data mapping, consistency, and standardization layer; *ii) *an integrative knowledge management layer to gather and integrate clinical and biomedical knowledge coming from various healthcare electronic health systems and research information systems, as well as public databases; and, *iii) *to build on top of the knowledge management layer, a qualitative and quantitative data exploitation layer with profile-specific visual data mining user-profiled interfaces.

In the standardization layer displayed at the bottom of Figure 3, mapping, consistency, and standardization of clinical data will primarily rely on in-place health information exchange (HIE) infrastructures, where standard terminology (e.g. SNOMED-CT, SERAM, SEMN, LOINC, etc.), message encoding (e.g. HL7 2.x / 3.x, MLHIM, openEHR, ISO 13606, etc.), message routing and Security (e.g. IPSec, Audit trail, Node authentication, etc.) will be used. Moreover, the HIE infrastructure will provide the required technical and syntactic interoperability to support the information exchange requirements inherent to the logics of integrated care processes. Specifically, the DHF considers the use of an open source Integrated Care Shared Knowledge Platform designed to support the execution of ICS, which allows to normalize the process logics of ICS to a common legacy schema or data model. Similarly, standardization of biomedical research data and metadata (ISA-Tab [13], MIAME, etc.) is a required task before its connection to the knowledge management layer. This step will not be required for those public biomedical research data sources already standardized (e.g. Ensembl, Uniprot and KEGG). The ICS Knowledge Platform also considers integration with personal health folders, so that valuable data generated outside the boundaries of formal healthcare institutions, such as self-management questionnaires and applications, genomic and biomedical data, will be gathered and effectively incorporated into the formal care arena in the context of an integrated care service prescribing interactions with patients via the personal health folder. Ultimately, data normalization and curation is the responsibility of each data source before interfacing with the knowledge management system. However, each data source should agree with the knowledge management layer how to effectively ensure data normalization. Finally, each data source should make use of their specific front-end interfacing to effectively support the normalization and curation of already existing data, or to support the normalization of the data right at the data source (e.g. questionnaires, lab tests, etc.).

Once all input data sources are properly encoded to a reduced set of common vocabularies and metadata schemas, and the exploitation rights granted, a knowledge management layer (as depicted in the middle of Figure 3) should allow the investigator to first integrate [14] and then to manage all of the above DHF-research information assets with easy to use and agile knowledge management graphical user web interfaces. The proprietary knowledge management solution assessed during the execution of the Synergy-COPD project [15] was based on the concept of knowledge as network by abstracting commonly used concepts and knowledge into objects and their relations. In addition, it bridged multiple sources and scales of knowledge and various deterministic and probabilistic models with a systems biology approach. Structuring explicit and implicit knowledge into these formal concepts enabled the use of existing well-defined vocabularies (e.g. GO, ICD10) and standards (e.g. SBML, HL7) to couple true semantic integration (i.e. the mapping of equivalent meaning and objects) across all information types relevant in translational research with a flexible and extensible data model, ensuring robustness against structural changes in services and data, transparent usage, and low set-up and maintenance requirements. This knowledge management layer may also facilitate non-technological aspects of human and cultural dimensions to effectively enable information and knowledge sharing.

Finally, this leads to the exploitation layer as displayed in the upper side of Figure 3 wherein potential use of profile-specific visual data exploitation portals are considered to support agile data querying at first and facilitating posterior data analytics. The latter will include a simulation environment capable of facilitating the user-friendly simulation of multi-scale predictive models, from common descriptive analytics to complex modelling approaches.

The user-profiled portals ought to be primarily used by the two research actors of the DHF, the clinical and the basic scientist (Table 1), allowing them, for example, to mine registered information to gain insight into mechanisms leading from health to disease transition and to study disease natural evolution, as well as interactions among diseases. Initial search, retrieval and R-plugin based data mining methods integrated into the Synergy-COPD knowledge base [16] enabled the retrieval of disease or case specific sub-networks by expert users but the validation showed that to enable application by clinical researchers a simplification of the user interface was required. To this end, the DHF-research considers the use of agile data querying interfaces such as those powered by the BioMart [17,18] platform, used in the international cancer genome consortium (ICGC) Data Portal [19]. However, connectors to the R programming language should be available to accommodate for the needs of basic researchers having a bioinformatics profile. In addition, standard data analytic tools commonly used by clinical researchers should be made available within the exploitation layer. Moreover, such user-profiled portals may include links to new tools such as the Synergy-COPD multi-scale simulation environment [20], designed to enable the explorative execution of computational models. Ultimately, this data exploitation process should enable the translation of novel biomedical research outcomes into real clinical practice via generation of rules feeding novel CDSS. The latter (CDSS) should be embedded into novel clinical processes defined in terms of integrated care services, so closing the DHF continuous improvement circle.

### Contributions of the DHF-research beyond the current state of the art

We performed a thorough review of available ICT platforms that have been designed to support biomedical research with a systems approach [21-25]. Unfortunately, the reported information is fragmented and often insufficient to display a complete picture of each translational research platform. We review here the main characteristics of five state of the art initiatives considering the above DHF-research functional layers, namely: data standardization, knowledge management and data exploitation.

***Standardization layer ***- All five systems [21-26] support integration of data encoded using standard terminologies for clinical data entities (e.g. ICD9-CM, SNOMED, LOINC, etc.), but such an extensive use of standard terminologies is more difficult to accomplish in the context of basic biomedical research data adding extra efforts for the appropriate integration of data from different omics levels (e.g. Ensemble database, based on the BioMart project [17,18]).

A completely differentiating issue is the potential to interoperate with in-place clinical information, as such a functionality was only reported in STRIDE [21] that showed interoperability through a Health Information Exchange (HIE) platform via HL7 RIM messaging. It is of note that none of the five systems analysed [21-25] showed potential to interoperate with chronic care clinical information as described above.

***Knowledge management layer ***- All platforms [21-25] have reported the use of multiple standard terminologies for semantic integration of data from various sources, but none of them describes a complete set of tools for inference analyses as reported in detail in [16]. There is also a clear need to extend the Open Access framework into the clinical and personal perspective.

***Exploitation layer ***- All state of the art translational research platforms offer comprehensive sets of data mining and exploitation tools through unique user portals targeting all types of researchers roles, which necessarily limits acceptability and usability of the system that otherwise could greatly improve with a user-profiled approach. Moreover, current data exploration and data querying tools (such as the i2b2 [23] query tool) show potential to evolve toward more dynamic and agile data exploration capabilities.

The review clearly identified the need for expanding the spectrum of users that should be achievable through a proper user-profiled orientation aiming at enhancing current user interfaces. An open source architecture with a service-oriented approach seems most adequate to build-up innovative business models allowing the continuous developments of enhanced functionalities based on sustainable costs-benefit ratios. Open Data schemas for all kind of data that the DHF integrates, the active role of patients and their empowerment to grant access to their own data, become crucial.

## Discussion

### Contributions to a biomedical research scenario for predictive medicine

The convergence of two major driving forces: *i) *current re-shaping of health systems seeking efficiencies through the alignment with the needs generated by chronic conditions, as discussed in detail in [1,27]), and, *ii) *rapidly evolving perspectives of biomedical knowledge [28] is fostering the transition towards a mature 4P medicine scenario for chronic patients. To the best of our knowledge, Synergy-COPD has provided one of the first relevant technological and biomedical contributions to this transition, using COPD and the analysis of co-morbidities as a use case. But most importantly, the project has identified important gaps, crucial unmet needs and strategic proposals that should help to consolidate the emerging scenario, specifically in the COPD case and in general to other disease areas. In this context, the conceptualization of the components of the DHF-research, as well as the proposals for its effective deployment together with the articulation of biomedical research with both formal and informal care, constitute one of the most significant outcomes of the project.

The deployment of the concepts described in the current report should allow an efficient bidirectional articulation between biomedical research and both healthcare and environmental factors with impact on disease development and activity. By doing so, we can reasonably speculate that preventive strategies modulating the transition from health to disease, as well as disease progress, will be effectively implemented and adopted as part of the future conventional health promotion and healthcare scenario. Finally, it is expected that such future healthcare scenario may influence business innovation in pharmacological industry and healthcare business models by overcoming current fragmentation among informal care, healthcare and biomedical research, which shall have a high impact in shortening time to market for both drug development and drug repositioning. It may also help to foster novel synergies between pharmacological and non-pharmacological therapies.

### Challenges for deployment of the DHF

The complexities of the deployment of the three-tier DHF, involving multiple interoperability levels (e.g. technological, organizational-cultural, etc.) and actors, are acknowledged.

*Informal Care *- Previous experiences using Personal Health Systems [29] in general and with the PHF-PDSS [7] in particular have shown the potential of this tool serving three main purposes: *i) *empowering citizens and patients toward an active role in disease prevention and management, *ii) *favouring collection of structured information on environmental, sociological and behavioural factors influencing health status, as well as informal care interventions, and, *iii) *contributing to organizational interoperability at health system level. However, practicalities limiting the extensive deployment and adoption of the PHF-PDSS have not been fully solved yet, but all of them seem actionable. These limiting factors encompass a wide spectrum of aspects, namely: technological approaches securing subject identification and data privacy, legal classification of the supporting systems as medical or non-medical devices, design of user-friendly interfaces and business models generating incentives for adoption, and also the interfacing between PHF-PDSS (informal care) and electronic health records (formal healthcare). It is of note, however, that all the above limiting factors are being addressed in several EU regions because the PHF-PDSS is becoming a pivotal component in the reshaping of the health system to face the challenge of chronic conditions.

*Formal care *- There is increasing evidence that deployment of articulated integrated care services (ICS) covering the entire spectrum of patient severity is a good strategy to enhance health outcomes with cost containment [30]. Different open ICT-platforms and strategies have been proposed to efficiently support the extensive deployment of ICS [30]. Implicit with those strategies are the integration of information collected through the PHF-PDSS (articulation with informal care) and clinical data normalization aiming at facilitating information sharing across healthcare tiers and among territories. Figure 3 in the current manuscript proposes such open ICT-platforms as a realistic option to integrate citizen information generated in either an informal or a formal care scenario, into biomedical research platforms (DHF-research). We acknowledge, however, that extensive deployment of those systems is still under development. A complementary approach to the proposal indicated in Figure 3 to facilitate short-term data integration into DHF-research could be the setting of highly curated clinical datasets specifically used for research purposes.

*Biomedical research *- The deployment of DHF-research proposals displayed in Figure 3 require stepwise implementation strategies wherein two key aspects clearly emerge as main short-term priorities. Firstly, ICT developments aiming at generating user-friendly portals for clinicians devoted to translational research. A second element is the convergence of on-going developments in the area of knowledge management giving particular priority to the assessment of inter-molecular (proteins, genes, or metabolites) interactions (physical or functional), transcriptional regulation and gene-disease association, that should foster the bridging between omics-generated knowledge and the clinical arena. Finally yet importantly, progresses in this area will be strongly associated to strategies fostering convergence of on-going ICT developments including both open and proprietary approaches. Finally, a relevant area is the design of innovative business models providing sustainability of biomedical research platforms beyond specific research and/or infrastructure projects that triggered the initial settings.

For all three DHF tiers, both legal and ethical aspects ensuring privacy and security of data transfer and management delineate a crucial area to be properly handled. Both data anonymization and encryption strategies have experienced a significant progress contributing to partially solving the problem. However, they show clear limitations in areas such as the management of genetic information wherein data anonymization is a challenge. In addition, the use of data for purposes other than the original data collection aims may generate limitations and activating communities of data donators for scientific purposes with a wider view than current practices is required. Clearly, evolving both legal frames and citizen cultural attitudes will be crucial to achieve a balance between social acceptance of anonymized data sharing together with reasonable levels of security and privacy of data transfer and management.

### Developments beyond the project lifetime

We fully acknowledge that the current manuscript only displays basic lessons learnt during the Synergy-COPD project life span. Next immediate steps to be taken before the end of the project are twofold. First, to establish a focus group, including all actors, for the qualitative assessment of the current DHF proposal. The aim of the focus group should be to perform a qualitative assessment of the DHF fitness to transfer the biomedical research use case of Synergy-COPD to more general clinical research needs. Second, to generate structured interactions with well-known international experts in the three tiers of the DHF to prioritize specific steps of the deployment strategy, and, to plan collaborative interactions with selected on-going projects addressing similar aims.

## Conclusions

The DHF is presented as a comprehensive and coherent ICT strategy supporting emerging requirements of applied systems medicine with novel interactions between informal care, formal healthcare and biomedical research. The current manuscript proposed ICT solutions for the three DHF tiers, extending more its biomedical research component, and stepwise strategies for effective deployment of the concept that should foster implementation of 4P medicine.

## Competing interests

DM is part of Biomax Informatics AG and DH is part of Almirall R&D. The rest of authors declare they have no competing interests.

## Authors' contributions

I. Cano, M. Lluch-Ariet, D. Gomez-Cabrero, Dieter Maier, S. G. Kalko, Marta Cascante, J. Tégner, F. Miralles and J. Roca made substantial contributions to conception and design of the Digital Health Framework; I. Cano, M. Lluch-Ariet, Dieter Maier and J. Roca participated in drafting the article; I. Cano, M. Lluch-Ariet, D. Gomez-Cabrero, Dieter Maier, S. G. Kalko, Marta Cascante, J. Tégner, F. Miralles, D. Herrera, J. Roca and the Synergy-COPD consortium revised the article critically for important intellectual content and gave final approval of the version to be submitted and any revised version.

## Declaration

Publication of this article has been funded by the Synergy-COPD European project (FP7-ICT-270086).

This article has been published as part of *Journal of Translational Medicine *Volume 12 Supplement 2, 2014: Systems medicine in chronic diseases: COPD as a use case. The full contents of the supplement are available online at http://www.translational-medicine.com/supplements/12/S2.
